# Probing the role of cation-π interaction in the thermotolerance and catalytic performance of endo-polygalacturonases

**DOI:** 10.1038/srep38413

**Published:** 2016-12-08

**Authors:** Tao Tu, Yeqing Li, Xiaoyun Su, Kun Meng, Rui Ma, Yuan Wang, Bin Yao, Zhemin Lin, Huiying Luo

**Affiliations:** 1 Key Laboratory for Feed Biotechnology of the Ministry of Agriculture, Feed Research Institute, Chinese Academy of Agricultural Sciences, No. 12 Zhongguancun South Street, Beijing 100081, P. R. China; 2 Institute of Animal Science and Veterinary Medicine, Hainan Academy of Agricultural Sciences, Haikou 571100, P. R. China

## Abstract

Understanding the dynamics of the key pectinase, polygalacturonase, and improving its thermotolerance and catalytic efficiency are of importance for the cost-competitive bioconversion of pectic materials. By combining structure analysis and molecular dynamics (MD) simulations, eight mutagenesis sites having the potential to form cation-π interactions were identified in the widely used fungal endo-polygalacturonase PG63. In comparison to the wild-type, three single mutants H58Y, T71Y and T304Y showed improved thermostability (the apparent *T*_*m*_s increased by 0.6−3.9 °C) and catalytic efficiency (by up to 32-fold). Chromatogram analysis of the hydrolysis products indicated that a larger amount of shorter sugars were released from the polygalacturonic acid by these three mutants than by the wild-type. MD analysis of the enzyme-substrate complexes illustrated that the mutants with introduced cation-π interaction have modified conformations of catalytic crevice, which provide an enviable environment for the catalytic process. Moreover, the lower plasticity of T3 loop 2 at the edge of the subsite tunnel appears to recruit the reducing ends of oligogalacturonide into the active site tunnel and initiates new hydrolysis reactions. This study demonstrates the importance of cation-π interaction in protein conformation and provides a realistic strategy to enhance the thermotolerance and catalytic performance of endo-polygalacturonases.

Plant cell walls are primarily composed of cellulose, hemicellulose, lignin, and pectin^1^. A promising industry for the production of second-generation biofuels is emerging with efforts to improve the enzymatic deconstruction of plant biomass into fermentable sugars^2^. Efficient bioconversion of this renewable, sustainable biomass has been receiving much attention due to the potential economic and environmental benefits of these biofuels. However, the biomass constituents used for biofuel production exclude pectin, and all bioconversion processes are mainly focused on the utilization of cellulose, hemicellulose, and lignin^3^,^4^. Pectin, which is synthesized in the secondary cell walls of the plant represents the bulk of plant materials^5^, is the most functionally and structurally complex polysaccharide and is found in the middle lamella of the primary cell walls^6^. Its presence accounts for the recalcitrance of the biomass and influences wood saccharification^7^,^8^. The pectin network consists of smooth and hairy regions, including smooth homogalacturonan (HG) and hairy rhamnogalacturonan-I (RG-I) and rhamnogalacturonan-II (RG-II). HG is composed of polymers of α-d-galacturonic acid (GalpA) residues linked at the *O*-1 and *O*-4 positions; RG-I contains repeated units of 1,4-α-d-galacturonic acid linked by α-L-rhamnosyl, branched α-L-arabinofuranosyl, and/or β-d-galactopyranosyl residues at the C-1 and C-2 positions^6^; and RG-II is smooth HG with methylated GalpA at *O*-6 and/or *O*-acetylation at C2 and/or C3.

Due to its complex structure, the complete breakdown of pectin by microorganisms requires the synergistic action of different enzymes. For instance, when phytopathogenic fungi attack plant cell walls, they first secrete pectin-degrading enzymes^9^. Of these enzymes, polygalacturonase (PG) is one of the most important virulence factors. Plants also produce PGs during the ripening process to soften and sweeten fruits^10^. According to the sequence homology and three-dimensional structures, PGs are confined to family 28 of the glycoside hydrolases (GH; http://www.cazy.org/GH28.html)^11^. Based on the action modes, PGs are grouped into endo-PGs (EC 3.2.1.15) and exo-PGs (EC 3.2.1.67, EC 3.2.1.82)^12^. Endo-PGs are the most well-characterized and studied pectinases that catalyze the hydrolysis of the α-1,4-glycosidic bonds between the polygalacturonic acid (PGA) at the smooth pectin regions, and thus are often preferred for technical applications, such as in fruit juice, feed, paper, and textile industries, as well as for the extraction of oils^13^. Therefore, a full understanding of the catalytic mechanism of endo-PGs is a key towards the engineering of new endo-PGs for various applications.

The members of the GH28 family share a conserved right-handed parallel β-helical structure of ten complete turns^14^,^15^. Shimizu *et al*.^16^ proposed that a carboxyl residue Asp173 acts as the proton donor and that two aspartates (Asp153 and Asp174) act as the general bases during the catalytic process of GH28 enzymes. The important roles of these three completely conserved and catalytically important aspartate residues in the endo-PGs have been verified using site-directed mutagenesis^17^. To date, three-dimensional structures of ten endo-PGs are available (http://www.rcsb.org). Although these endo-PGs demonstrate similar three-dimensional structures, they vary in specificities, catalytic activity, pH preference, and thermostability. Most fungal endo-PGs have an optimal temperature of 20–60 °C, retaining stability at temperatures 50 °C or below; are active at an acidic to neutral pH (2.5–6.0); and vary in catalytic activities by up to five orders of magnitude (1–28,000 U/mg, http://www.brenda.uni-koeln.de).

The catalytic process of enzymes includes 1) ligand binding to the active site of the enzyme; 2) hydrolysis of the glycoside bond; and 3) product release^18^. During this process, the enzyme molecule experiences different conformations of the enzyme-substrate complex and rapid transition to support maximum activity^19^. This dynamic state is controlled by the balance of weak interactions of hydrogen bonds, salt bridges, and hydrophobic effects^20^,^21^. Among them, cation-π interaction has been revealed to play a critical role in stabilizing protein structure^22^. In this study, we first elucidated the important roles of cation-π interaction in the catalytic process of endo-PG. By introducing a cation-π interaction into the widely used GH28 endo-PG, PG63, from *Penicillium* sp. CGMCC 1669^23^, three single mutants were constructed, and their thermostability and catalytic performances were compared with that of the wild type. This study deepens the understanding of the importance of this non-covalent interaction in enzyme catalysis and provides a novel strategy to improve GHs for greater catalytic performance.

## Results

### Identification of mutagenesis sites in PG63 to form the cation-π interaction

**Figure 1:**
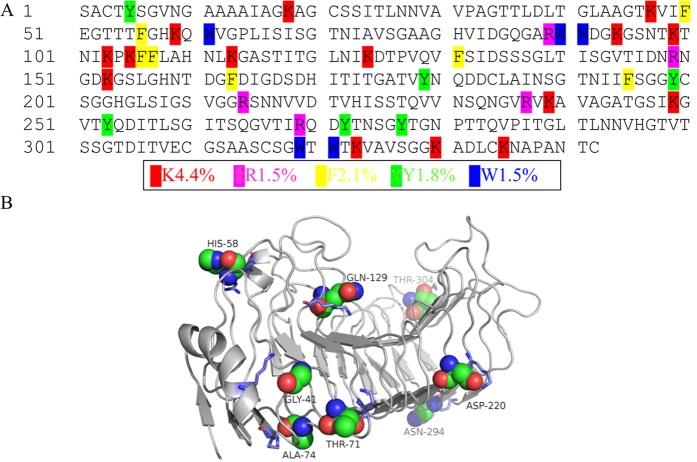
Selection of mutagenesis sites in endo-polygalacturonase PG63 of *Penicillium* sp. (**A**) The amino acid sequence of deduced PG63 with colored residues (Lys, Arg, Phe, Tyr and Trp) that have potentials to form cation-π interactions. (**B**) The homology-modeled PG63 (shown from the N-terminus). The mutation sites that form cation-π interactions with an occupancy rate of >50% after 10 ns MD simulations were selected.

Based on the primary sequence of PG63 (342 residues, Fig. 1A), amino acids that are likely involved in the formation of the cation-π interaction were identified, including 15 Lys, 7 Phe, 6 Tyr, 5 Arg, and 5 Trp residues (38 in total). Because Lys and Tyr lead to favorable cation-π interactions in thermophilic proteins^24^, residues in close proximity to the 38 targets of modeled PG63 were substituted to Lys or Tyr *in silico*. As a result, 43 mutants were obtained *in silico* (Additional file 1). On this basis, the brute-force approach combined with 10-ns molecular dynamics (MD) simulations was used to examine the occupancy rate during the trajectory of each potential cation-π interaction. The possibility of each mutant forming a cation-π interaction was assessed according to two rules: 1, the distance cutoff between the mass center of the positively charged group and aromatic residue containing the π-system should be less than or equal to 6 Å, and 2, the absolute value of the dihedral angle defined between the side chain of two residues should be less than or equal to 60°. In combination with the MD simulation result, only eight mutants have an occupancy rate of >50% (Additional file 1). These potential mutagenesis sites, i.e. G41Y, H58Y, T71Y, A74Y, Q129Y, D220Y, N294Y, and T304Y, are all located on the protein surface and far from the catalytic center except for Gln129 (as shown in Fig. 1B). Substitution of these residues is presumed to have no direct effect on catalytic efficiency, but probably makes an indirect impact through non-covalent interactions.

### Experimental characterization of selected mutants

To test the effect of cation-π interaction on enzymatic properties, the eight mutants described above were successfully constructed by site-directed mutagenesis. The wild-type PG63 and its mutants were then produced in *P. pastoris* GS115 and secreted into the culture media. The aim of this study is to find out whether and how cation-π interaction can affect the catalytic performance of the enzyme, and the specific activity of each enzyme towards PGA (pH 4.0, 50 °C and 10 min) were first compared. Among the eight mutants, mutants H58Y, T304Y and T71Y exhibited significantly higher specific activities than wild-type PG63 (14,900 ± 838, 2,600 ± 67, and 1,900 ± 59 U/mg vs. 1,600 ± 51 U/mg, respectively). Therefore, the triple mutant H58Y/T71Y/T304Y was produced as described above and had a specific activity of 5,100 ± 204 U/mg under the same conditions, indicating that cation-π interaction at the specific site contributes to the improvement of catalytic efficiency; however, more cation-π interactions have no additive effects. The reason might be that more cation-π interactions mitigate the adjacent interaction network by competition (e.g., as with a salt bridge)^25^.

**Figure 2:**
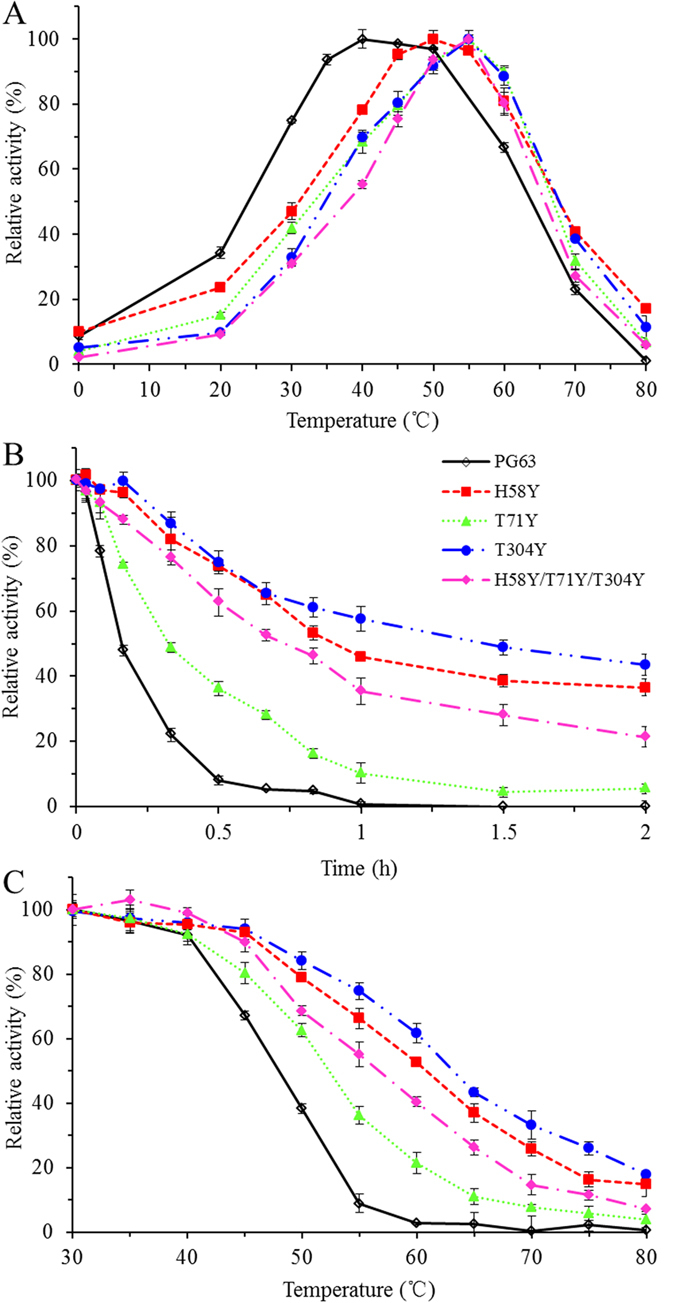
The thermal properties of wild-type PG63 and its mutants. (**A**) Temperature-dependent activity profiles determined at pH 4.0 for 10 min. (**B**) Time courses of thermal inactivation of each enzyme at pH 4.0 and 55 °C. (**C**) Enzyme inactivation assay at different temperatures for 30 min. Each value is shown as means ± standard deviations (n = 3).

The introduction of these aromatic residues had no effect on the functional pH but distinctively influenced the functional temperature. The wild-type PG63 and its four mutants (H58Y, T71Y, T304Y and H58Y/T71Y/T304Y) exhibited optimal activities at pH 4.0 to 4.5 (data not shown), suggesting that the introduced aromatic residues were mis-sense protons through cation-π interactions. The temperatures for maximum enzymatic activities (*T*_max_) of mutants H58Y, T71Y, T304Y, and H58Y/T71Y/T304Y were 50, 55, 55 and 55 °C, respectively (Fig. 2A), 10–15 °C higher than that of wild-type PG63 (40 °C), although the wild-type PG63 showed 97% of the maximal activity at 50 °C. To further investigate the thermal properties of the four mutants, their half-lives (*t*_1/2_) at 55 °C and thermodynamic stabilities (*T*_m_) were compared with that of wild-type PG63. In comparison with the *t*_1/2_ value (9.6 min) and *T*_*m*_ value (56.0 °C) of wild-type PG63, mutants H58Y, T71Y, T304Y and H58Y/T71Y/T304Y had improved thermostability, i.e. longer half-lives (19.3−86.4 min, Fig. 2B) and increased apparent *T*_*m*_s (~3.9 °C, 0.6 °C, 3.4 °C, and 2.6 °C, respectively). As shown in Fig. 2C, the *T*_50_ value of wild-type PG63 was determined to be 48 °C, while the mutants H58Y, T71Y, T304Y, and H58Y/T71Y/T304Y had increased *T*_50_ values of 13 °C, 3 °C, 16 °C, and 9 °C, respectively.

The kinetic parameters of wild-type PG63 and its four mutants were determined using PGA as the substrate at pH 4.0 and 50 °C (Table 1). Compared to wild-type PG63, mutants H58Y, T71Y, T304Y, and H58Y/T71Y/T304Y showed increased substrate affinity (*K*_*m*_, 1.21−3.66 mg/mL vs. 4.43 mg/mL), catalytic rate (*k*_*cat*_, 1.53−10.52 × 10^3^/s vs. 1.16 × 10^3^/s), and decreased energy (Δ(ΔG), −0.65−[−10.13] kJ/mol), leading to a ~0.25−32.19-fold increase in catalytic efficiency (*k*_*cat*_/*K*_*m*_). Located on the protein surface and far from the catalytic center (Fig. 1B), these mutations only changed the local structures (Additional file 2) but significantly influenced both substrate binding and catalysis.

### Isothermal-titration calorimetry (ITC) assay

To study the substrate binding mechanism, inactive wild-type PG63 and its three single mutants were each constructed by replacing the catalytic Asp182 with Asn^16^. Using GalpA3 as the substrate, the thermodynamics of enzyme-substrate were determined using the ITC. The raw binding curves (Additional file 3) all fit well to the independent binding models, with a calculated value (stoichiometry) of *n* = 1 ± 0.08. As shown in Table 2, all mutants had stronger affinity to GalpA3 than wild-type PG63, with an increased *K*_*a*_ value (4.20−5.19 × 10^2^ M^−1^ vs. 3.34 × 10^2^ M^−1^). The substrate binding of all enzymes is enthalpy-driven, as the enthalpy values (Δ*H*) are all negative. The highly favorable Δ*H* values offset the negative entropy values (Δ*S*) and result in negative ΔG^0^ values. Compared to the wild-type PG63 (−3.51 kcal/mol), the three single mutants showed increased ΔG^0^ values of −2.97, −0.16, and −1.52 kcal/mol, respectively. These ΔG^0^ increases might be ascribed to the interaction strength between substrate and enzyme and the changes in conformational entropy^26^. These results are consistent with the decreased *K*_m_ values.

### Hydrolysis product analysis

**Figure 3:**
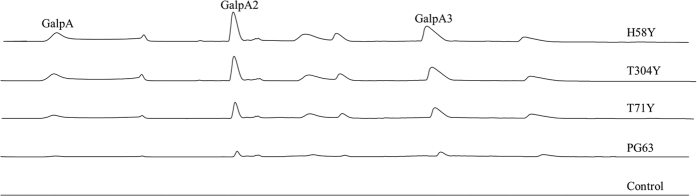
Chromatograms of the hydrolysis products of PGA.

Wild-type PG63 hydrolyzed PGA into small oligogalacturonides in an endo-action mode^23^. The hydrolysis profiles of PGA by wild-type PG63 and its three single mutants showed a similar mode of action but varied in hydrolysis efficiencies (Fig. 3). After incubation with PGA for 60 min, the main hydrolysis products by wild-type PG63 were GalpA2 and GalpA3. However, a larger amount of shorter sugars (i.e. GalpA, GalpA2 and GalpA3) were detected in the three single mutants (H58Y, T71Y and T304Y). Among them, mutant H58Y had the highest hydrolytic activity than mutants T71Y and T304Y.

### Molecular docking

**Figure 4:**
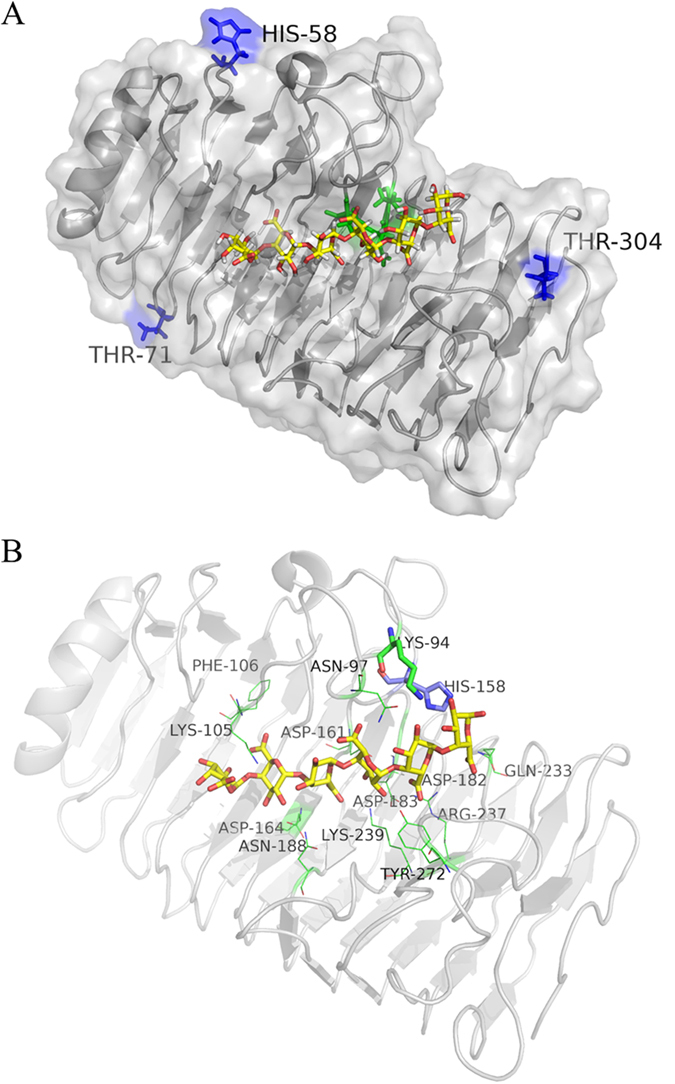
Structural analysis of the interactions of PG63-GalpA6 complex. (**A**) Modeled PG63 bound to GalpA6. The catalytic residues and mutation sites are shown in green and blue, respectively. (**B**) Close view of GalpA6 in the catalytic pocket of PG63 and of the tunnel formed due to the interaction of side groups of amino acid residues.

To elucidate the underlying mechanisms of cation-π interaction in endo-PG thermotolerance and catalysis improvement, the GalpA6 was docked into the substrate-binding pocket of wild-type PG63, followed by minimization. Based on the active-site architecture of galacturonate units co-crystallized with *Stereum purpureum* endo-PG I (1KCD) (Additional file 4), which authorizes efficient contacts of the GalpA6 with the catalytic residues, the wild-type PG63 binds to GalpA6 along the −4 to +2 subsites, yielding one GalpA2 and one GalpA4 as the hydrolysis products (Fig. 4A). In addition to the conserved side chains of Asn97, residues Arg237, Lys239, and Tyr272 are all involved in binding GalpA at +1 subsite. The atom NE2 of His150 of *S. purpureum* endo-PG I (1KCD, corresponding to His158 in PG63) forms a hydrogen bond with the O2 hydroxy group of GalpA at +1 subsite^16^. However, the side chain of His158 is disordered and does not interact with GalpA6. 158HNTD161 (PG63 numbering) is one of the four strictly conserved sequence segments in fungal PGs^9^; therefore, the residue His158 probably has an evolutionary relationship with the functions of the enzyme.

### MD simulation

**Figure 5:**
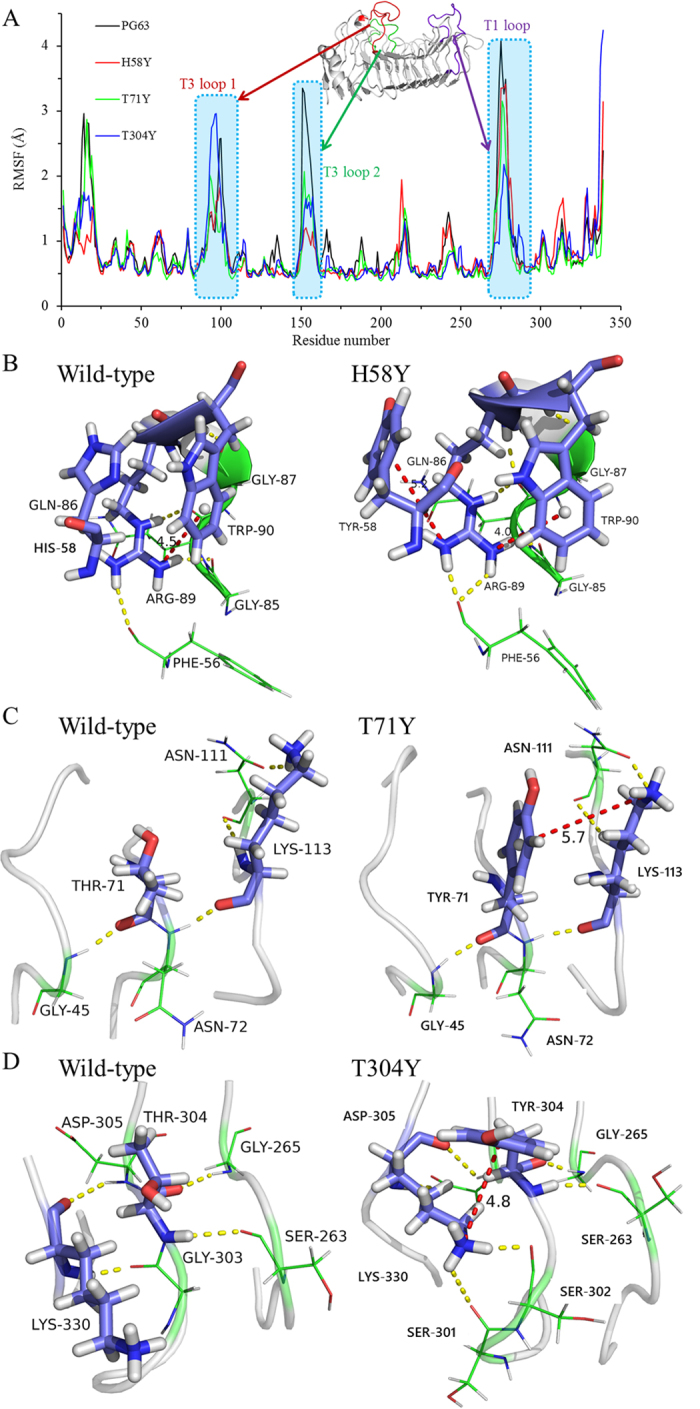
Flexibility analysis of wild-type PG63 and its mutants. (**A**) RMSF analysis of the mobility profiles of enzyme-substrate complexes relative to the first conformation blocks. Each system was run twice. (**B–D**) Interaction regions accounting for the different mobility of wild-type PG63 and mutants H58Y, T71Y and T304Y, respectively.

In order to gain insights into the substrate binding in the catalytic crevice of the enzyme, the wild type enzyme-substrate complex was submitted to *in silico* mutagenesis with PyMOL. During the course of these simulations, the root mean square deviation (RMSD) plots of the α-carbon atoms of four systems (wild-type PG63 and its three single mutants) showed stable plateaus after 30 ns (Additional file 5), indicating that each system maintains equilibrium within the last 20 ns. Meanwhile, the plasticity of wild-type PG63 and its three single mutants was evaluated by root mean square fluctuations (RMSF) analysis through two independent runs (Fig. 5A). A few regions, especially the loops between β-sheets, displayed significant differences in mobility. For instance, the T3 loops 1 (residue 90−106) and 2 (residue 147−162) of mutants H58Y and T71Y exhibit lower mobility relative to that of wild-type PG63, and the T3 loop 2 (residue 147−162) and T1 loop (residue 269−289) in wild-type PG63 are more flexible than those in mutant T304Y. These local effects caused subtle differences and could have altered the interactions between some specific residues, as depicted in Fig. 5B–D. In mutant H58Y (Fig. 5B), one of the positively charged groups in the side chain of Arg89, i.e. the NH2 atom, participates in the formation of a cation-π interaction with Trp90 (4.0 Å), and another NH1 atom forms a cation-π interaction with Tyr58 (5.2 Å). This double dentate mode appears to play an important role in stabilizing the protein. Furthermore, the Phe56 located on the T3 loop interacts with Arg89 via two hydrogen bonds in mutant H58Y (one more than that in wild-type PG63), restricting the mobility of the T3 loop in the N-terminus. In mutant T71Y (Fig. 5C), residues Tyr71 and Arg113 form a cation-π interaction (5.7 Å) that is located on the two adjacent loops. Meanwhile, four hydrogen bonds are identified as in wild-type PG63, which stabilizes the three loop regions. Similarly, residue Tyr304 interacts with Lys330 through a cation-π interaction (4.8 Å), and two more hydrogen bonds (S301@O/K330@NZ and S302@O/K330@NZ) are identified in mutant T304Y than in wild-type PG63 (Fig. 5D). Located on the reducing end of the active site, where a long loop freely swings, these bonds contribute to stabilizing the last turn of the loop.

**Figure 6:**
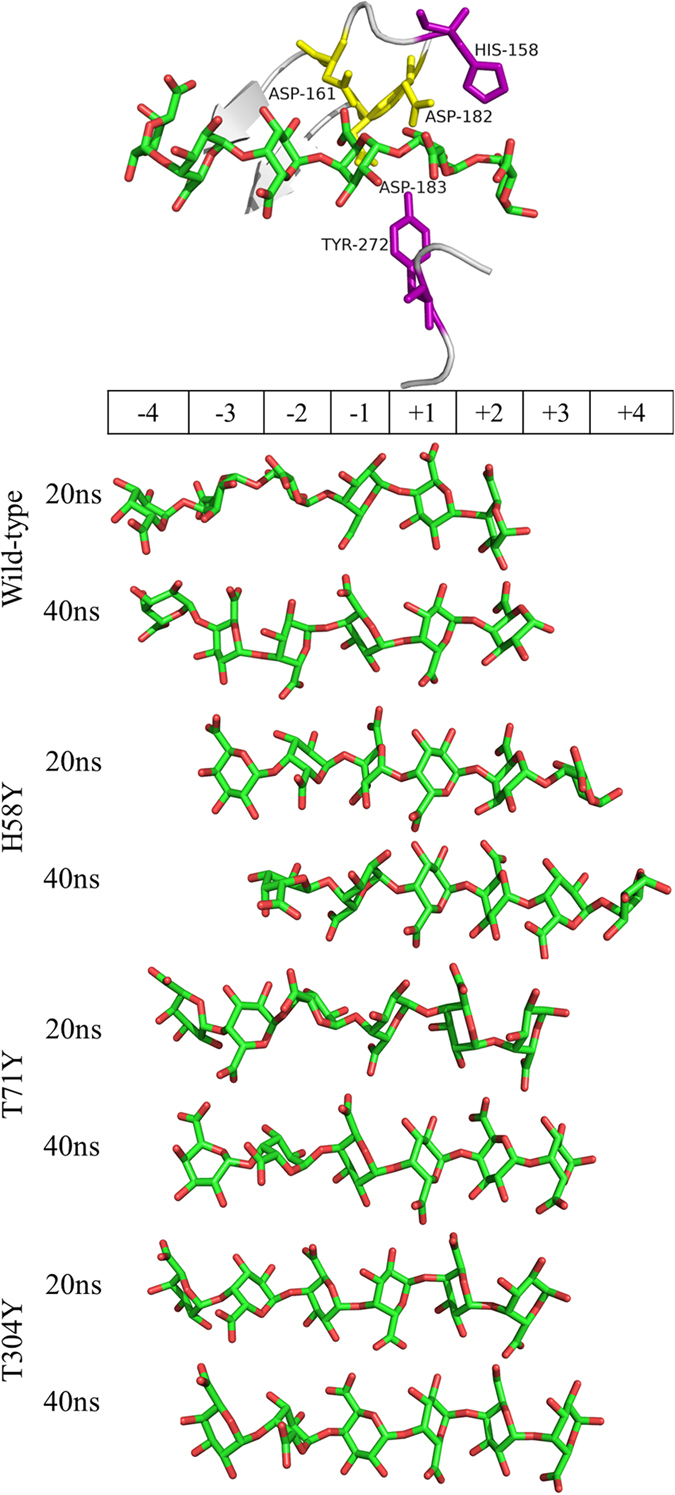
MD simulations of the substrate sliding along the catalytic cleft of wild-type PG63 and its mutants. Snapshots were taken at two different stages along the complete simulation. The GalpA6 was placed along the schematic binding groove with the catalytic residues at the subsite −1/+1 (top panel). The wild-type PG63 and its mutants had similar starting complex conformations that positioned along the −4 to +2 subsites. The simulations were performed twice for each enzyme.

The MD trajectories also provide valuable information on the interactions between substrate and enzyme. It is worth noting that the T3 loop 2 region (residue 147−162) has much lower plasticity in all single mutants than in wild-type PG63 (Fig. 5A). The rigidity of the T3 loop 2 region follows the order of mutant H58Y > T304Y > T71Y > wild-type PG63, which is consistent with the catalytic activity of the enzymes. Hence, the catalytic crevices of the enzymes were compared with each other. The resolved endo-PG’s crystal structure shows that His158 and Tyr272 are located at the two sides of the entrance of the catalytic cleft and function primarily in substrate recognition^16^,^17^. The computed GalpA6 trajectories were inspected regularly, as shown in Fig. 6. The GalpA6 is positioned corresponding to the catalytic triad of wild-type PG63 along the −4/+2 subsites, and remains in a state of equilibrium to enable catalysis during the simulation. In contrast, the three single mutant systems show that GalpA6 starts to slide from the binding cleft at about 18 ns, although it remains connected to the active-site residues. During the final simulation stage of mutant H58Y, the conformation of GalpA6 in the crevice was displaced by two binding sites with respect to its starting position, to evolve from −4/+2 to −2/+4 subsites. A similar phenomenon occurred in mutants T71Y and T304Y systems with one binding site to evolve from −4/+2 to −3/+3 subsites.

## Discussion

The noncovalent cation-π interaction relevant to structural biology and other diverse roles has been gaining increasing attentions. For example, cation-π interactions are well known to have a stabilizing role in the secondary, tertiary, and quaternary structure of proteins through electrostatic interactions^24^,^27^. The Arg residues scattered over the surface of the fibronectin III-like (FnIII) domain of a β-glucosidase play an important role in the interaction with lignin by means of cation-π stacking with the aromatic rings^28^. Additionally, the aromatic side chain of Tyr74 in the amino acid antiporter AdiC is a critical pH sensor that is mediated by cation-π interaction^29^. However, the contribution is currently unknown with respect to cation-π interaction’s role in the catalytic performance of enzymes. Our goal herein is thus to probe the role of cation-π interaction in catalysis by introducing cation-π interaction into the widely used GH28 endo-PG, PG63, from *Penicillium* sp. CGMCC 1669.

The intramolecular cation-π interaction is a well-known factor responsible for protein thermostability. For example, our study is in agreement with other studies that thermostability can be significantly enhanced by the cation-π interaction between Lys202 and Trp204 of an alkaline α-amylase from *Alkalimonas amylolytica* and Lys7 and Trp99 of a tyrosinase from *Streptomyces kathirae* SC-1^30^,^31^. The double dentate mode of cation-π interactions (Arg89-Trp90 and Arg89-Tyr58) in mutant H58Y, the cation-π interaction Arg113-Tyr71 located on the two adjacent loops in mutant T71Y, and the cation-π interaction Lys330-Tyr304 stabilizing the C-terminal long loop freely swings in mutant T304Y showed a significant increase in apparent *T*_*m*_ value of ~3.9 °C, 0.6 °C, and 3.4 °C, respectively. In summary, these conformational shifts within the active loop may account for the increased thermostability of three mutants. On the other hand, the thermal performance of the mutants follows the order of wild-type PG63 < T71Y < T304Y < H58Y and corresponds to their occupancy rates (54.8%, 81.9%, and 83.0%; Additional file 1). This indicates that the computational method we used is reliable and can provide decisive guidance in designing proteins for greater thermotolerance.

Theoretically, the amounts of reducing ends generated from PGA hydrolysis after treatment with the same amount of enzymes should be the same. However, significant differences were observed between wild-type PG63 and its mutants (Fig. 3). A larger amount of small oligogalacturonides were released from PGA by the three single mutants than by the wild-type. That is, wild-type PG63 failed to degrade relatively longer oligogalacturonides, whereas the three mutants had this capacity. These results are also supported by the profound changes of *k*_*cat*_/*K*_*m*_ of the three single mutants on the PGA (Table 1). However, this action mode is far from processivity due to the similar product ratios of GalpA2 and GalpA3. Beyond that, processivity is dependent on an arginine residue at a position commensurate with subsite −5 in PG^32^. PG63 has a serine residue at this position and thus functions in a non-processive mode. The mutants’ capacity to degrade relatively longer oligogalacturonides is thought to be critical to improving the catalytic efficiency of substrate hydrolysis^33^.

For the MD trajectories, GalpA6 can move along the catalytic crevice without completely leaving the cleft, which helps catalytic residues immediately initiate continuous hydrolysis. Based on the hydrolysis profiles of the products, the mutants hydrolyzed more relatively long oligogalacturonides to GalpA, GalpA2, and GalpA3. These results suggest that the introduction of a cation-π interaction may modify the conformation of the catalytic crevice and provide an enviable environment for catalytic processes. Given the findings described above, the authors assume that the lower plasticity of T3 loop 2 at the edge of the subsite tunnel recruits the reducing ends of oligogalacturonide into the active site tunnel and initiates new hydrolysis reactions. It is widely acknowledged that some critical residues can stack with the ring faces of sugar units, forming carbohydrate-π interactions, and play a vital role in enzyme-substrate recognition. For instance, three residues (Tyr7, Trp23, and Tyr112) constitute a harboring wall to contact the substrate through hydrophobic interactions as they occurred in the endoglucanase 3 from *Trichoderma harzianum* did^34^. In the wild-type PG63-GalpA6 complex, the side chain of strictly conserved His158 located on the T3 loop 2 region is disordered and observed to have different conformations; however, in the mutant complexes it converges into a single conformation. The plasticity of residue His158 is much lower in mutant enzymes by introducing a cation-π interaction than in wild-type PG63. The residue His158, located on the T3 loop 2 region, is more flexible in wild-type PG63 than those mutants, and is unable to act as a director fixed on the entrance of the catalytic cleft. From this perspective, it is similar to processivity in which the substrate binds in a tunnel, with multiple attacks occurring on the single carbohydrate chain^32^, although the three mutant enzymes are typically nonprocessive enzymes as the amounts of GalpA, GalpA2, and GalpA3 increase simultaneously. In other words, the importance of the conserved His158 located at T3 loop 2 might be comparable to that of Trp40 of cellobiohydrolase TrCel7A, the key residue to load chain ends and to initiate continuous hydrolysis^35^.

## Conclusion

The thermotolerance and catalytic efficiency of the widely used endo-PG are remarkably improved through introduction of a cation-π interaction. This bond might restrict the mobility of the active loop and modify the conformation of the catalytic crevice, thus providing an enviable environment for catalytic processes. As a result, this engineering approach enhanced the efficiency of substrate degradation and the production of oligogalacturonides of lower polymerization degrees. This study reveals the significance of cation-π interaction in protein conformation and provides a realistic strategy to enhance the catalytic performance of proteins.

## Methods

### Plasmids, strains, culture media and materials

The recombinant plasmid used in this work was pPIC9-*pg63*, which contained the gene *pg63* (HQ446162) from the strain *Penicillium* sp. CGMCC 1669^23^. Site-directed mutagenesis was carried out using the specific primers (Additional file 6) and two-step polymerase chain reactions (PCRs). All mutants were verified by double-stranded plasmid sequencing. *Escherichia coli* Trans I-T1 (TransGen, Beijing, China) and *P. pastoris* GS115 (Invitrogen, Carlsbad, CA) were used for plasmid amplification and heterologous expression, respectively. The pEASY-T3 vector (TransGen) and pPIC9 (Invitrogen) were used for plasmid construction, respectively. Culture media for *His*^+^ transformants selection and *P. pastoris* growth and induction were prepared according to the manual of the *Pichia* Expression kit (Invitrogen). Mono-, di-, and trigalacturonic acid (GalpA, GalpA2 and GalpA3) and the substrate PGA were purchased from Sigma-Aldrich (St. Louis, MO). All other standard chemicals were of analytical grade.

### Identification of potential mutagenesis sites

Theoretically, when the cationic side chain of lysine or arginine is in close proximity to the side chain of an aromatic residue (tryptophan, tyrosine, or phenylalanine), the geometry is biased toward the formation of a favorable cation-π interaction^36^. Moreover, the side chains of electron-rich tryptophan, tyrosine, and phenylalanine can engage in a variety of supramolecular interactions^20^. Hence, the potential targets (Lys, Arg, Phe, Trp, and Tyr) involved in the formation of a cation-π interaction were identified in the deduced protein sequence of PG63. The CaPTURE program^36^ and a brute-force approach were used to assess the possibility of each residue substitution to form a cation-π interaction with one of the candidate targets identified above. Then, the 10-ns MD simulations were carried out with an AMBER 14 simulation package for further screening, and the occupancy rate during the trajectory of each putative cation-π interaction was calculated. The mutants that formed a cation-π interaction with an occupancy rate of >50% were selected for site-directed mutagenesis.

### Protein preparation

Wild-type PG63, eight single mutants (G41Y, H58Y, T71Y, A74Y, Q129Y, D220Y, N294Y, and T304Y) and one triple mutant (H58Y/T71Y/T304Y) were produced in *P. pastoris* as described previously^23^. The cell-free culture supernatants were collected by centrifugation, followed by concentration using Vivaflow 200 ultrafiltration membranes with a 5-kDa molecular mass cutoff (Vivascience, Hannover, Germany), desalting in 20-mM McIlvaine buffer (pH 4.0) on a HiTrap desalting column (GE Healthcare, Uppsala, Sweden), and chromatographic analysis on a HiTrap Q XL FPLC column (GE Healthcare). All enzymes were purified to greater than 95% homogeneity as determined by sodium dodecyl sulfate-polyacrylamide gel electrophoresis (SDS-PAGE), with a 5% stacking gel and a 12% separation gel.

### Enzymatic activity assay and biochemical characterization

The endo-PG activity was determined by measuring the release of reducing sugars from PGA using the 3,5-dinitrosalicylic acid (DNS) method with d-(+)-GalpA as the standard, as described previously^37^,^38^. One unit of endo-PG activity was defined as the amount of enzyme that released reducing sugars equivalent to 1 μmol of d-(+)-GalpA per minute under assay conditions (pH 4.0, 50 °C and 10 min). All reactions were repeated three times. The protein concentrations were determined using the Bio-Rad protein assay kit (Boston, MA).

The pH-activity profiles of wild-type PG63 and its mutants were determined at 50 °C for 10 min in 100-mM McIlvaine buffer (pH 3.0–8.0). The optimal temperature was determined by performing the same process at temperatures ranging from 0 to 80 °C in 100-mM McIlvaine buffer (pH 4.0) for 10 min. The enzyme half-lives (*t*_1/2_) were determined by measuring the residual activities under standard conditions after various durations of incubation at 55 °C without substrate. For the determination of *T*_50_ (a temperature at which 50% of the maximal activity of an enzyme is retained), the enzymes (50 μg/mL) were incubated at a temperature range of 30 to 80 °C for 30 min without substrate, and the residual activities were then measured at standard conditions.

The Michaelis constants (*K*_*m*_) and kinetic constants (*k*_*cat*_) of wild-type PG63 and its mutants were determined under standard conditions (pH 4.0, 50 °C, and 5 min) with PGA of 0.5 to 10 mg/mL as the substrate. The kinetic values were calculated by fitting the Lineweaver–Burk plot. All reactions were performed in triplicate.

### Differential-scanning calorimetry (DSC) assay

The thermodynamic stabilities (*T*_*m*_, melting temperature) of wild-type PG63 and its mutants were determined on a MicroCal™ VP-Capillary DSC (GE Healthcare) as described previously^13^. Aliquots of proteins (0.35 mg) were dissolved in 1 mL of 20-mM McIlvaine buffer (pH 4.0) and loaded onto the capillary automatically. The melting temperature, *T*_*m*_, corresponded to the maximum of the transition peak over 20–90 °C within a heating and scanning rate of 120 °C/h. The test was repeated at least twice. The same McIlvaine buffer was used as the blank control.

### ITC assay

ITC was employed to measure the binding affinities between wild-type PG63 or the three single mutants with GalpA3 at 25 °C using an ITC 200 microcalorimeter (GE Healthcare, Waukesha, WI)^39^. To avoid a catalysis reaction in the titration experiment, site-directed mutagenesis was conducted to obtain inactive wild-type PG63 and its three single mutants by replacing the catalytic residue Asp182 with Asn by overlap PCR with specific primers (Additional file 6). Protein expression and purification were conducted as described above. Each protein was adjusted to the final concentration of 30 μM in McIlvaine buffer (pH 4.0). The oligomer at the desired concentration in McIlvaine buffer (pH 4.0) was injected into the protein cell 20 times with the stirring speed set to 1000 rpm. The titration of the ligand with the buffer served as the control. The association constant *K*_*a*_ was calculated accordingly using the software MicroCal Origin 7.0.

### Hydrolysis product analysis

The capacities of wild-type PG63 and its mutants to hydrolyze PGA were evaluated using high-performance anion-exchange chromatography (HPAEC; Thermo Fisher Scientific, Sunnyvale, CA) as described previously^40^. Purified recombinant proteins (0.1 U) were incubated with 3 mg of PGA in McIlvaine buffer (pH 4.0) at 50 °C for 60 min. Samples (200 μL) were extracted regularly, heated in a bath of boiling water for 5 min to inactivate the enzymes, and centrifuged at 12,000 × *g* at 4 °C for 10 min. Each sample (25 μL) was injected into a high-performance anion exchange chromatography (HPAEC) system model 2500 equipped with a CarboPac PA200 column (3 × 250 mm). The oligosaccharides were eluted by NaOH (0.2–1 M) at a flow rate of 0.45 mL/min. GalpA, GalpA2, and GalpA3 were used as the standards.

### Homology modeling, molecular docking and molecular dynamic simulation

The homology modeling of wild-type PG63 was built based on the X-ray structure of endo-PG from *Colletotrichum lupini* (2IQ7_A, 61% identity) by using the Swiss Model server. The structure was optimized (especially loops) for energy minimization by YASARA software (www.yasara.org) to remove steric clashes. PyMOL (Delano Scientific, Portland, OR) was applied to visualize and analyze the generated models.

AutoDock Vina was selected as the docking tool to determine enzyme- and substrate-binding conformation according to the user’s guide^18^,^41^. Wild-type PG63 was used as the receptor, and the substrate hexagalacturonic acid (GalpA6) coordinates of the X-ray crystal structure of a pectin methylesterase in complex with GalpA6 (2NTB) was used as ligand. The C_α_ atom of Lys239, which is an important contributor for recognizing the carboxyl group of the substrate, was selected as the center of the box. A docking grid with a size of 60 Å × 60 Å × 60 Å was used to encompass the ligand partial-protein complex. The set number generated for the docking poses was 20. The best binding conformation was selected by the criteria of interacting energy combined with the geometrical matching quality and the binding affinity (ΔG, kcal/mol) of the AutoDock Vina software. After that, energy minimization was used to relax atomic clashes for the complex using YASARA software^3^,^13^. This process ultimately obtained a reliable model for the PG63-GalpA6 complex.

The simulations were carried out with the AMBER 14 simulation package and repeated twice for each system. The standard AMBER force field ff99SB and GLYCAM_06j-1 were used to generate the topologies and parameters of the enzyme and substrate, respectively^42^,^43^. Each system was immersed in a dodecahedral periodic box of TIP3P water molecules that extended 10 Å from the protein atoms^44^. Six sodium ions were added to maintain the system’s neutrality. Before the MD simulations, the water molecules/ions were resolved to minimize energy via 1,000 steps, followed by 20,000 steps for the side chains of the protein, and then 4,000 steps for the whole system to remove potentially poor contacts between the solute and solvent. After energy minimization, the systems were gradually heated from 0 to 300 K over 100 ps, followed by 50-ns production of MD simulations with a time step of 2 fs at a temperature of 300 K and pressure of 1.0 atm that were controlled by the Langevin algorithm^45^. Long-range electrostatic interactions were treated using the particle-mesh Ewald (PME) method^46^. Bonds involving hydrogen atoms were constrained by the SHAKE algorithm^47^.

## Additional Information

**How to cite this article**: Tu, T. *et al*. Probing the role of cation-π interaction in the thermotolerance and catalytic performance of endo-polygalacturonases. *Sci. Rep.* **6**, 38413; doi: 10.1038/srep38413 (2016).

**Publisher's note:** Springer Nature remains neutral with regard to jurisdictional claims in published maps and institutional affiliations.

## Supplementary Material

Supplementary Information

## Figures and Tables

**Table 1:** Kinetic values of wild type PG63 and its mutants^a^.

Enzymes	*K*_*m*_ (mg/mL)	*k*_*cat*_^b^ (×10^3^/s)	*k*_*cat*_/*K*_*m*_ (×10^2^ mL/mg/s)	Δ(ΔG)^c^ (kJ/mol)
Wild type	4.43 ± 0.15	1.16 ± 0.03	2.62 ± 0.15	0
H58Y	1.21 ± 0.06	10.52 ± 0.06	86.97 ± 4.04	−10.13
T71Y	3.66 ± 0.11	1.20 ± 0.02	3.28 ± 0.05	−0.65
T304Y	2.83 ± 0.09	1.53 ± 0.03	5.41 ± 0.27	−2.10
H58Y/T71Y/T304Y	2.12 ± 0.10	3.10 ± 0.03	14.63 ± 0.79	−4.97

^a^The kinetic values are shown as means ± standard deviations (n = 3).

^b^The *k*_*cat*_ values were calculated on the hypothesis that the enzymes are in monomeric forms.

^c^Δ(ΔG) = −RT·ln[(*k*_*cat*_/*K*_*m*_)_mut_/(*k*_*cat*_/*K*_*m*_)_wt_], where (*k*_*cat*_/*K*_*m*_)_mut_ and (*k*_*cat*_/*K*_*m*_)_wt_ are the *k*_*cat*_/*K*_*m*_ ratios of the mutant and wild type enzymes, respectively, R is the ideal gas constant, and T is the temperature in Kelvin.

**Table 2:** Thermodynamic parameters of wild-type PG63 and its three single mutants determined by ITC^a^.

Enzymes	*k*_*a*_ (×10^2^ M^−1^)	Δ*H* (kcal/mol)	Δ*S* (cal/mol/K)	ΔG^0^^b^ (kcal/mol)	Δ(ΔG^0^) (kcal/mol)
Wild type	3.34 ± 0.16	−102.20 ± 2.78	−331	−3.51	0
H58Y	5.19 ± 0.12	−97.71 ± 3.72	−306	−6.48	−2.97
T71Y	4.20 ± 0.13	−87.15 ± 1.47	−280	−3.67	−0.16
T304Y	4.94 ± 0.06	−74.50 ± 0.67	−233	−5.03	−1.52

^a^Experiments were performed at 25 °C in McIlvaine buffer (pH 4.0).

^b^Gibbs free energy, ΔG^0^, is calculated using the mathematical equation ΔG^0^ = Δ*H*−TΔ*S*, where T is the absolute temperature in Kelvin.
