# Supplementary material for: Probing the role of cation-π interaction in the thermotolerance and catalytic performance of endo-polygalacturonases
Source: Sci Rep. 2016 Dec 8;6:38413. doi: 10.1038/srep38413 (PMC5143973; doi:10.1038/srep38413)
Supplement: Supplementary Information [file srep38413-s1.doc]

**Additional files to**

**Probing the role of cation-π interaction in the thermotolerance and catalytic performance of endo-polygalacturonase**

Tao Tu^1^, Yeqing Li^1^, Xiaoyun Su^1^, Kun Meng^1^, Rui Ma^1^, Yuan Wang^1^, Bin Yao^1^, Zhemin Lin^2^ & Huiying Luo^1^

^1^Key Laboratory for Feed Biotechnology of the Ministry of Agriculture, Feed Research Institute, Chinese Academy of Agricultural Sciences, No. 12 Zhongguancun South Street, Beijing 100081, P. R. China. ^2^ Institute of Animal Science and Veterinary Medicine, Hainan Academy of Agricultural Sciences, Haikou 571100, P. R. China. Correspondence and requests for materials should be addressed to Z.L. (email: Lzmin01@126.com) or H.L. (email: luohuiying@caas.cn).

**Additional file 1: The occupancy rate during the trajectory of each potential cation-π interaction introduced into the *Penicillium* sp. PG63 *in silico*.^a^**

| **Candidate target** | **Selected site** | **Occupancy rate (%)** | **Candidate target** | **Selected site** | **Occupancy rate (%)** |
| --- | --- | --- | --- | --- | --- |
| Y5 | A13K^b^ | 31.7 |  | V82Y | 39.5 |
|  | A16K | 1.2 | F131 | F107K | 25.8 |
| K18 | D38Y | 15.2 | K153 | N150Y | 0.3 |
|  | G41Y | 51.2 | F163 | I165K | 46.0 |
| K47 | A74Y | 55.0 | F195 | I173K | 41.8 |
| F50 | L26K | 45.8 |  | I224K | 9.0 |
| F56 | L65K | 41.7 | Y199 | Q228K | 25.4 |
| K59 | A33Y | 1.0 |  | T177K1 | 33.0 |
| R89 | H58Y | 83.0 | R214 | N188Y | 5.1 |
| W91 | A88K | 18.0 | K249 | D220Y | 54.6 |
|  | L156K | 46.2 | Y253 | I208K | 0.0 |
| K94 | N97Y | 3.5 |  | V236K | 47.3 |
|  | T98Y | 7.9 | R269 | E309Y | 46.6 |
| K99 | D92Y | 11.3 |  | S275Y | 2.1 |
| K103 | V62Y | 34.0 | Y277 | P281K | 5.6 |
| K105 | Q129Y | 65.5 | W319 | L290K | 0.2 |
|  | S132Y | 49.2 |  | V308K | 28.3 |
| F106 | V130K | 0.4 | W321 | L292K | 44.2 |
| F107 | I66K | 0.5 |  | L306K | 21.4 |
| K113 | T71Y | 54.8 | K323 | N294Y | 67.6 |
|  | S137Y | 0.1 | K330 | T304Y | 81.9 |
| K124 | D147Y | 4.0 |  |  |  |

^a^ The occupancy rate was calculated by splitting the corresponding MD trajectory into 10 ns under two conditions: 1) the mass center of each positive charge group is located at a distance of ≤ 6 Å distance cutoff to that of aromatic residue containing the π-system; 2) the dihedral angle between the side chains of two residues is ≤ 60°.

^b^ Lys or Tyr was selected for residue substitution due to their favorable formation of cation-π interaction in thermophiles proteins (Gromiha et al., 2002). The mutation sites with occupancy rate higher than 50% are highlighted in yellow.

**Additional file 2: Comparison of the local structures of wild-type PG63 (A, C, E) and mutants H58Y (B), T71Y (D) and T341Y (F).**


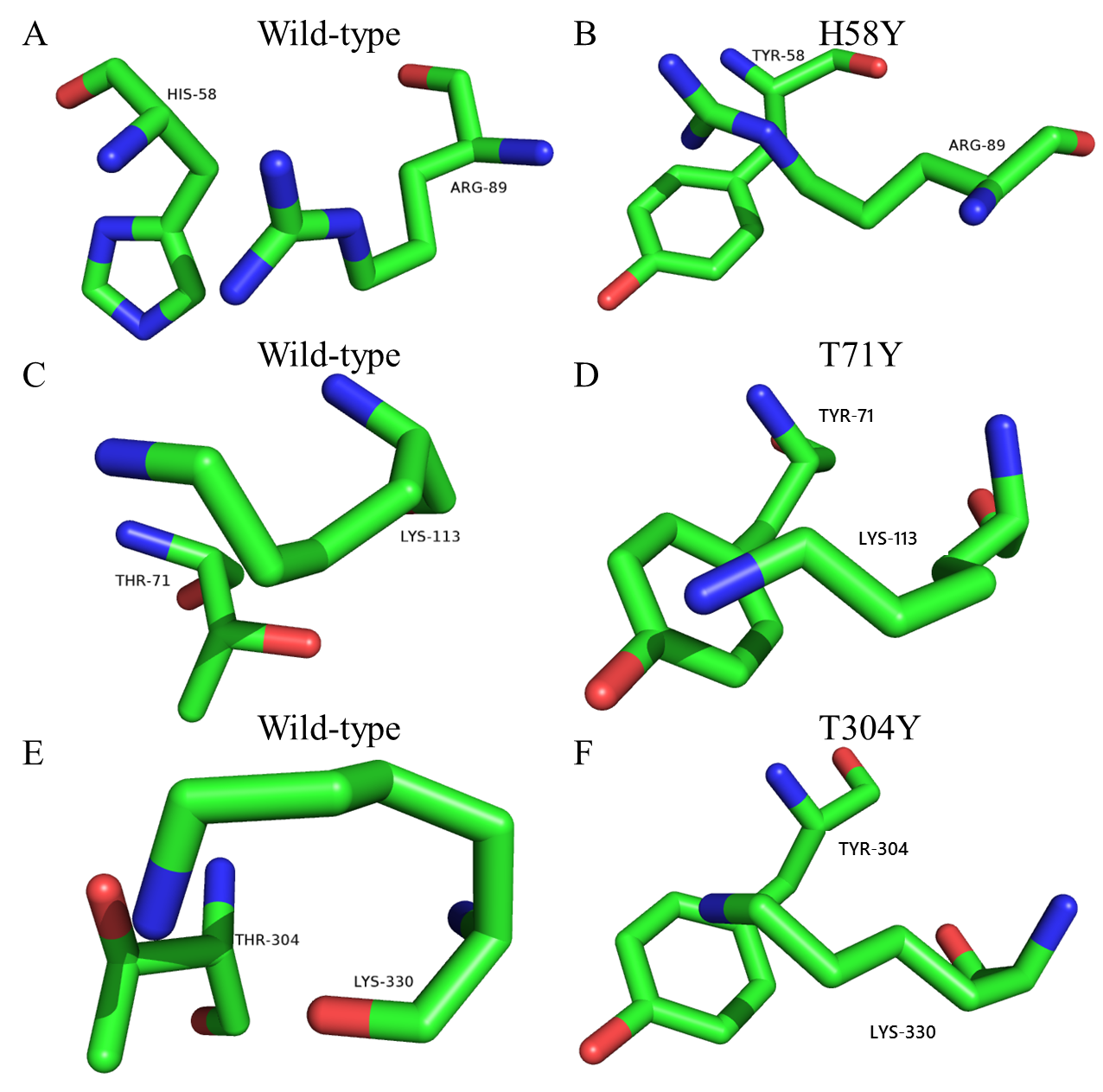


**Additional file 3: ITC analysis of the binding isotherms (lower panels) and thermograms (upper panels) of inactive wild-type PG63 and its three single mutants towards GalpA3.** Binding experiments were performed at 25°C in 25 mM McIlvaine buffer (pH 4.0) in triplicate.


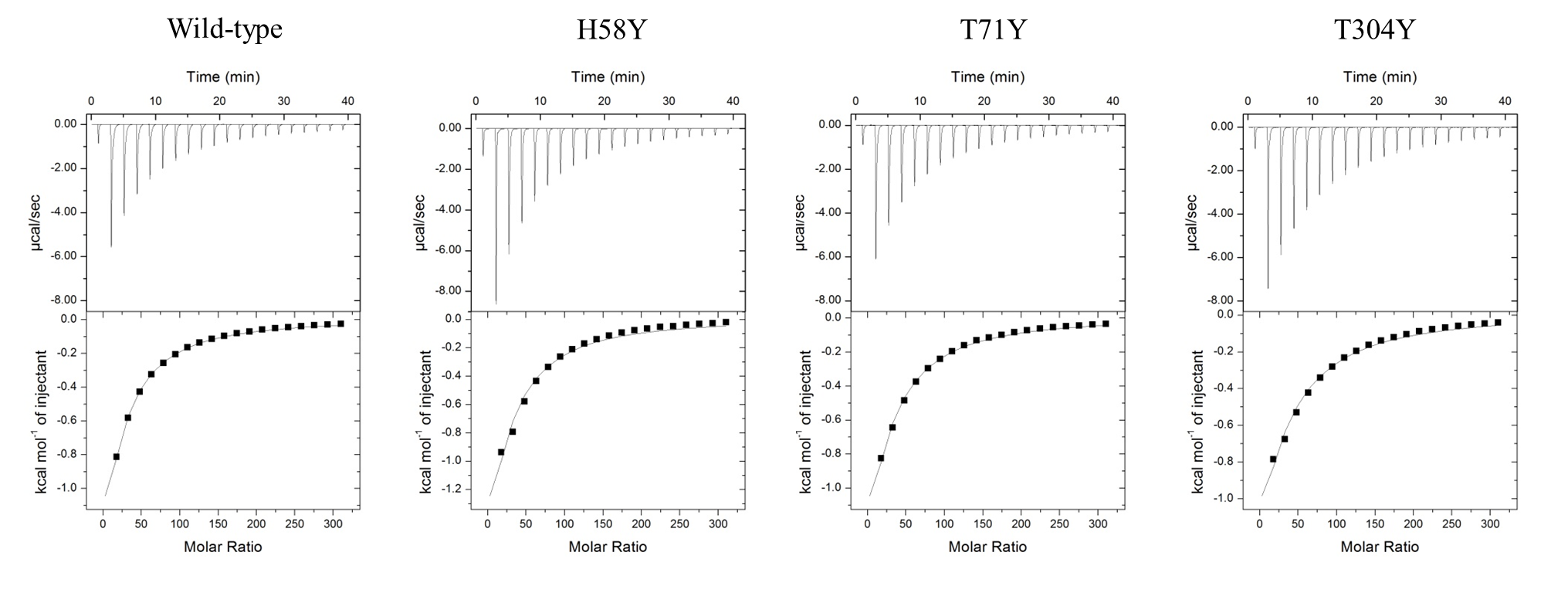


**Additional file 4: Docking of GalpA6 with wild-type PG63.** The coordinate of GalpA6 was obtained from the crystal structure of [a pectin methylesterase in complex with GalpA6](http://www.ncbi.nlm.nih.gov/Structure/mmdb/mmdbsrv.cgi?uid=59149) (2NTB). The only endo-PG-galacturonate complex (1KCD) known so far was used as the template to assess the reliability of homology-modeled enzyme-substrate complex constructed in the present study. The two well superimposed conformations of GalpA at +1 subsite mean reasonable modeling of the wild-type PG63-GalpA6 complex.


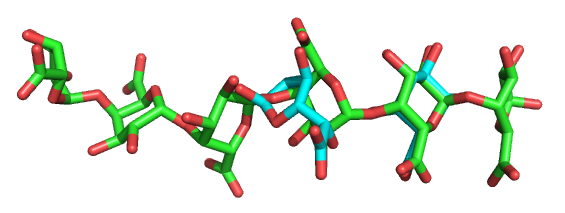


−4 −3 −2 −1 +1 +2

**Additional file 5: RMSD analysis of the trajectories of wild-type PG63 and its three single mutants with Amber at 300 K during a 50-ns MD simulation.**

**
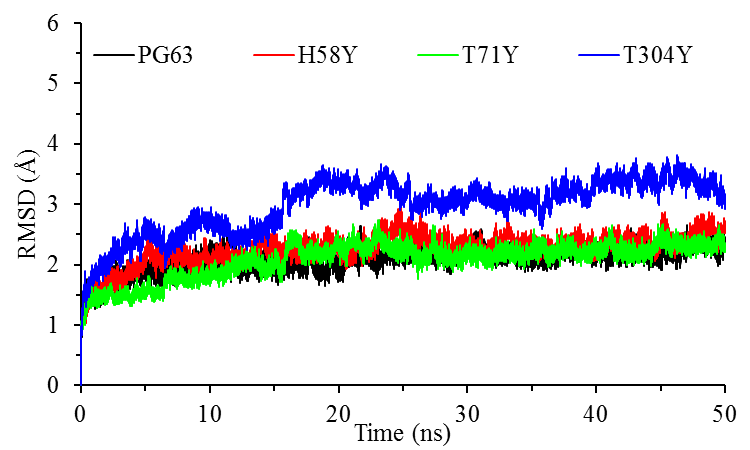
**

**Additional file 6: Primers used in this study.**

| Primer | Oligonucleotide sequence (5′→3′)*^a^* |
| --- | --- |
| G41Y-F | GCTGGATTTGACTTACCTGGCCGCGGGT |
| G41Y-R | GTACCCGCGGCCAGGTAAGTCAAATCCAG |
| H58Y-F | CACTACTTTCGGCTACAAGCAGTGGGTGGG |
| H58Y-R | GCCCACCCACTGCTTGTAGCCGAAAGTAGT |
| T71Y-F | TCTCCATCTCTGGGTACAACATCGCAGTTT |
| T71Y-R | CAGAAACTGCGATGTTGTACCCAGAGATG |
| A74Y-F | TCTGGGACCAACATCTACGTTTCTGGGGCT |
| A74Y-R | GCAGCCCCAGAAACGTAGATGTTGGTCCC |
| Q129Y-F | GGATACTCCCGTTTACGTCTTCAGCATCG |
| Q129Y-R | TCGATGCTGAAGACGTAAACGGGAGTATCC |
| D220Y-F | TCCAATAATGTGGTATACACCGTCCATATCAGC |
| D220Y-R | GCTGATATGGACGGTGTATACCACATTATTGG |
| N294Y-F | GACTCACCTTGAATTACGTGCACGGCAC |
| N294Y-R | ACCGTGCCGTGCACGTAATTCAAGGTGA |
| T304Y-F | GTCACATCCAGTGGCTACGATATCACCGTC |
| T304Y-R | CTCGACGGTGATATCGTAGCCACTGGATGT |
| D182N-F | CAGTTTATAACCAAAACGACTGCCTGGCCATC |
| D182N-R | TGGCCAGGCAGTCGTTTTGGTTATAAACTG |

*^a^*The mutation sites are underlined.
